# Static and Dynamic Electronic (Hyper)polarizabilities of Dimethylnaphthalene Isomers: Characterization of Spatial Contributions by Density Analysis

**DOI:** 10.1155/2013/832682

**Published:** 2013-10-28

**Authors:** Andrea Alparone

**Affiliations:** Department of Chemistry, University of Catania, Viale A. Doria 6, 95125 Catania, Italy

## Abstract

Static and frequency-dependent electronic (hyper)polarizabilities of the dimethylnaphthalene (DMN) isomers were computed in vacuum using the Coulomb-attenuating Density Functional Theory method. The nonlinear optical Second Harmonic Generation (SHG) and Electro-Optical Pockels Effect (EOPE) were investigated at the characteristic Nd:YAG laser wavelength of 1064 nm. The response electric properties especially the longitudinal polarizability, polarizability anisotropy, and first-order hyperpolarizability are significantly affected by the position of the methyl groups. The SHG and EOPE techniques can be potentially useful to discriminate the *α*,*α*-DMN isomers (2,6-DMN < 2,7-DMN < 2,3-DMN) as well as the *β*,*β*-DMN isomers (1,5-DMN < 1,4-DMN < 1,8-DMN). The (hyper)polarizability differences among the investigated DMNs were elucidated through density analysis calculations. The predicted polarizabilities exhibit good linear relationships with the experimental first-order biomass-normalized rate coefficient, a physicochemical property connected to the rates of biodegradation processes of polycyclic aromatic hydrocarbons.

## 1. Introduction

**Figure 1:**
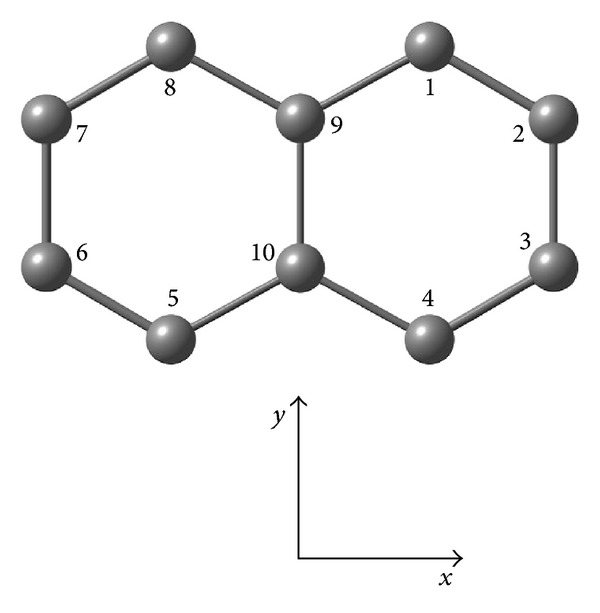
Coordinate system and atom numbering of dimethylnaphthalene (DMN) isomers.

Polycyclic aromatic hydrocarbons (PAHs) are very stable organic lipophilic pollutants principally produced during incomplete combustion processes, often exhibiting high levels of toxicity, mutagenicity, and carcinogenicity to humans and/or other living creatures [1–5]. Since PAHs are ubiquitous contaminants, there is much interest in searching practical strategies for their identification and removal from environments. Generally, alkylated derivatives are found in prevalence in the environmental samples of PAHs [6–8]. Dimethylnaphthalenes (DMNs) are substituted PAHs of great environmental interest [9–15]. DMNs can exist as ten isomeric forms (Figure 1): 1,2-DMN, 1,3-DMN, 1,4-DMN, 1,5-DMN, 1,6-DMN, 1,7-DMN, 1,8-DMN, 2,3-DMN, 2,6-DMN, and 2,7-DMN. It has been previously recognized that the enzymatic biodegradation of DMNs in aqueous media is strongly affected by the specific position of the methyl substituents [12]. Indeed the first-order biomass-normalized rate coefficient (*k*_*b*_) of DMNs varies within one order of magnitude, the *β*,*β*-disubstituted isomers showing the maximum *k*_*b*_ values along the series [12]. Therefore, the detection of the DMN isomers in the environmental mixtures is of fundamental importance from environmental and biochemical viewpoints. On the basis of experimental and theoretical investigations, the active site of the enzyme which controls the biodegradative mechanism is mainly characterized by hydrophobic residues [12, 13], involving contributions from dispersive and/or inductive forces in enzyme-substrate complex formation. This result has been corroborated by recent computational studies on the electronic polarizabilities (*α*) [14, 16] and Raman spectra [15] of alkylated-naphthalenes, the average polarizabilities [14], and summation of the Raman activities [15] of DMNs being found to be linearly related to the experimental *k*_*b*_ values. However, although the average polarizabilities (〈*α*〉) of DMNs regularly increase in the order 〈*α*〉(*α*,*α*-DMNs) < 〈*α*〉(*α*,*β*-DMNs) < 〈*α*〉(*β*,*β*-DMNs), they slightly vary among the ten isomers (up to 3.5%). Thus, the average polarizability is little useful to discriminate unambiguously the DMN isomers. A physicochemical property much more affected by the structural features is the electronic first-order hyperpolarizability (*β*) and the related nonlinear optical (NLO) properties Second Harmonic Generation (SHG) and Electro-Optical Pockels Effect (EOPE) [17–32]. SHG measurements are currently employed for the structural identification of biomolecules such as peptides and proteins [28–32].

The main focus of this study is to determine the static and frequency-dependent electronic *α* and *β* values of the series of the DMN isomers, aiming to explore the effects of the position of the CH_3_ groups on these electric properties, potentially helpful for the isomeric discrimination. The electronic (hyper)polarizabilities, are commonly predicted by means of *ab initio* and/or Density Functional Theory (DFT) computations. However, as well-known in the literature for an accurate determination of the electronic (hyper)polarizabilities, the choice of the functional is critical, especially in the case of *π*-conjugated compounds [33]. In fact, on the whole, the conventional DFT methods tend to systematically overestimate the electronic (hyper)polarizabilities obtained by high-level correlated *ab initio* levels. On the other hand, the long-range corrected DFT methods incorporating nonlocal effects [34, 35], describe adequately the diffuse regions of the charge distributions, giving much more satisfactory performances for the prediction of the response electric properties. In the present study we used the Coulomb-attenuating hybrid exchange-correlation functional (CAM-B3LYP) [36], which has been recently employed with success for computing electronic (hyper)polarizabilities of organic compounds [37–48].

## 2. Computational Details

All calculations were performed with the Gaussian 09 program [49]. We used the molecular geometries previously optimized at the DFT-B3LYP level with the 6-31G* basis set [14]. Static and frequency-dependent electronic (hyper)polarizability tensor components *α*_*ij*_ and *β*_*ijj*_  (*i*, *j* = *x*, *y*, *z*) were obtained through the Coupled-Perturbed Hartree-Fock procedure [50, 51] using the DFT-CAM-B3LYP method with the polarized and diffuse 6-31+G*  basis set. The CAM-B3LYP functional and the 6-31+G*  basis set can be considered suitable choices especially for the prediction of the electronic (hyper)polarizabilities of organic molecules [37–48, 52–54]. However, we checked the performances of the basis set (6-31+G*  versus POL Sadlej's basis set [55]) and level of calculation (CAM-B3LYP versus second-order Møller-Plesset perturbation theory (MP2)) on the related compound toluene. Dynamic (hyper)polarizabilities were evaluated at the experimental Nd:YAG laser wavelength of 1064 nm (*ℏω* = 0.04282 a.u.) for the SHG [*β*(−2*ω*; *ω*, *ω*)] and EOPE [*β*(−*ω*; *ω*, 0)] NLO processes. 

We report the dipole moments (*μ*), the isotropically averaged polarizabilities (〈*α*〉), the polarizability anisotropies (Δ*α*), and the isotropically first-order hyperpolarizabilities (*β*_*vec*⁡_), which are defined, respectively, as [56]

(1) μ=μx2+μy2+μz2,〈α〉=13(αxx+αyy+αzz),Δα={12[(αxx−αyy)2+(αxx−αzz)2+(αyy−αzz)2    + 6(αxy2+αxz2+αyz2)]}1/2,βvec⁡=βx2+βy2+βz2,

where *β*_*i*_  (*i* = *x*, *y*, *z*) is given by *β*_*i*_ = (1/3)∑_*j*=*x*,*y*,*z*_(*β*_*ijj*_ + *β*_*jij*_ + *β*_*jji*_).

## 3. Results and Discussion

### 3.1. Basis Set and Level of Calculation Effects: Response Electric Properties of Toluene

For an accurate prediction of the electronic (hyper)polarizabilities, the choice of the basis set and level of computation is of crucial importance [57–67]. In the present study we explored the effects of the basis set and theoretical level on toluene as a test case.  Table 1 reports the 〈*α*〉, Δ*α*, and *β*_*vec*⁡_ values of toluene calculated using the CAM-B3LYP and MP2 levels with the 6-31+G* and Sadlej's POL basis set. The latter basis set was specifically constructed for polarizability computations and has been recently employed with success to predict the electronic polarizabilities of naphthalene (N) [68]. However, it is well-demonstrated that for *π*-conjugated compounds the smaller 6-31+G* basis set furnishes an adequate alternative to the POL as well as more extended basis sets for predicting response electric properties, but at significantly minor computational costs [39, 45–48, 52–54].

The present results show that when passing from the 6-31+G* to the POL basis set, only marginal effects are observed. In fact, the 〈*α*〉 and Δ*α* values increase by 0.75 Å^3^ (+6.5%) and 0.25 Å^3^ (+3.9%), respectively, whereas the *β*_*vec*⁡_ decreases by 37.3 × 10^−53^ C^3^m^3^J^−2^ (−13.3%). Note that the (hyper)polarizability calculations carried out using the 6-31+G* basis set require noticeably minor CPU resources than those with the POL basis set (by about a factor of twenty!). In addition, we investigated the effects of the computational method, by comparing the CAM-B3LYP and MP2 (hyper)polarizability data. In line with the recent literature [37–39, 45–48, 69], the differences between the two levels are further smaller than those found for the basis sets, the 〈*α*〉, Δ*α*, and *β*_*vec*⁡_ values being calculated within 0.07 Å^3^ (0.6%), 0.19 Å^3^ (3.0%), and 4.2 × 10^−53^ C^3^m^3^J^−2^ (1.3%), respectively. Thus, considering the above results, the CAM-B3LYP/6-31+G* level can be judged as an acceptable compromise between accuracy and computational cost and has been entirely employed for the subsequent calculations on the static and frequency-dependent (hyper)polarizabilities of the DMN isomers. 

### 3.2. Static and Dynamic Polarizabilities of the DMN Isomers

Table 2 lists the static and frequency-dependent polarizabilities of the DMNs calculated in the gas phase at the CAM-B3LYP/6-31+G* level. For all the isomers, *α*_*xx*_ is the largest component, giving 43–49% of the total polarizabilities (*α*_*xx*_ + *α*_*yy*_ + *α*_*zz*_). The dispersion effects here evaluated at the *ℏω* = 0.04282 a.u. are rather modest, increasing the static *α*_*xx*_, 〈*α*〉 and Δ*α* values by 0.54–0.70 Å^3^ + (2%), 0.34–0.36 Å^3^ (+2%) and 0.40–0.55 Å^3^ (+3%), respectively. Table 2 also reports the data of the unsubstituted compound N for which some experimental and high-level correlated *ab initio* values are available in the literature [68, 70]. The static CAM-B3LYP/6-31+G*  *α*_*xx*_, 〈*α*〉 and Δ*α* values of N agree satisfactorily with both the observed (within −0.8, −2.8, and +2.3%, resp.) [70] and CCSD/POL data (within −2.0, −4.0, and +2.4%, resp.) [68]. Not surprisingly, the static CAM-B3LYP/6-31+G*〈*α*〉 values of the DMN isomers underestimate the previously calculated B3LYP/6-31+G* figures [14] by 0.42–0.51 Å^3^ (2.0–2.4%), principally owing to the introduction of nonlocal effects.

The order of the static and dynamic CAM-B3LYP/6-31+G*α_*xx*_ values is the following:

 1,4-DMN ~ 1,5-DMN < 1,8-DMN < 1,2-DMN < 1,3-DMN ~ 1,6-DMN < 1,7-DMN < 2,3-DMN < 2,6-DMN ~ 2,7-DMN.

For the 〈*α*〉 values, the above order is slightly modified, with the 1,8-DMN and 2,6-DMN isomers being, respectively, the less and more polarizable along the series:

 1,8-DMN < 1,4-DMN ~ 1,5-DMN < 1,2-DMN < 1,7-DMN ~ 1,3-DMN ~ 1,6-DMN < 2,3-DMN < 2,7-DMN ~ 2,6-DMN.

The order of the Δ*α* values is rather similar to that found for the *α*_*xx*_ data, except for the inversions between 1,6-DMN and 1,7-DMN and between 2,6-DMN and 2,7-DMN:

 1,4-DMN < 1,5-DMN < 1,8-DMN < 1,2-DMN ~ 1,3-DMN ~ 1,7-DMN < 1,6-DMN < 2,3-DMN < 2,7-DMN < 2,6-DMN.

**Figure 2:**
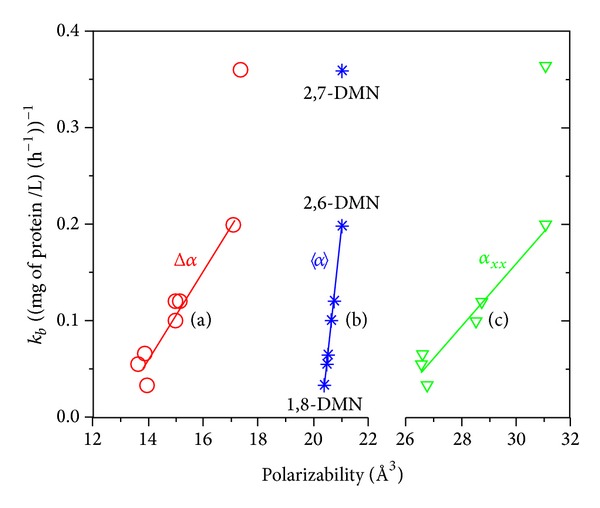
Relationships between the experimental biomass-normalized first-order rate coefficient *k*_*b*_ [12] and the gas phase CAM-B3LYP/6-31+G* polarizabilities of the DMN isomers. (a) *k*_*b*_ = −0.591 + 0.046 × Δ*α*  (*r* = 0.97); (b) *k*_*b*_ = −5.245 + 0.258 × 〈*α*〉  (*r* = 1.00); (c) *k*_*b*_ = −0.826 + 0.033 × *α*_*xx*_  (*r* = 0.97).

All the predicted polarizabilities concordantly increase on passing from the *α*,*α*-DMNs to the *α*,*β*-DMNs and then to the *β*,*β*-DMNs, in agreement with the previous 〈*α*〉 data obtained at the B3LYP/6-31+G* level in gaseous and aqueous phases [14]. Specifically, when we compare the 1,4-DMN and 2,6-DMN isomers, the static CAM-B3LYP/6-31+G* *α*_*xx*_, 〈*α*〉 and Δ*α* values enhance by 4.51 Å^3^ (+17.0%), 0.53 Å^3^ (+2.6%), and 3.32 Å^3^ (+24.1%), respectively. However, whereas the static 〈*α*〉 data slightly change along the series of isomers (within 0.65 Å^3^, 3.2%), the Δ*α* and *α*_*xx*_ values are distributed over larger ranges being within 3.32 Å^3^ (24.1%) and 4.54 Å^3^ (17.1%), respectively. On the whole, these results suggest that, in comparison to the average polarizabilities, the *α*_*xx*_ and Δ*α* properties are much more affected by the position of the methyl substituent, being potentially useful to identify the DMN isomers. Additionally, in agreement with a previous study on the average polarizabilities [14], the present *α*_*xx*_ and Δ*α* data of DMNs are found to be linearly related to the biodegradation experimental biomass-normalized first-order rate coefficients *k*_*b*_ [12], confirming the crucial role of the polarizabilities in the biodegradation process of this group of organic pollutants. The *α*_*xx*_/*k*_*b*_, 〈*α*〉/*k*_*b*_, and Δ*α*/*k*_*b*_ linear relationships are displayed in Figure 2 showing good statistics since the *r* value is predicted between 0.97 and 1.00 (following the discussion reported in [14], the 2,7-DMN isomer was excluded from the relationships).

In order to explain the polarizability differences among the DMN isomers, we analyzed the spatial contributions of electrons to the polarizabilities by using the concept of density of polarizability *ρ*_*j*_^(1)^(*r*) [71, 72]. The *ρ*_*j*_^(1)^(*r*) is defined as derivative of the charge density function *ρ*(*r*, *F*) with respect to the applied fields *F* (*r* is the position vector). The *ρ*(*r*, *F*) is commonly expanded in powers of *F* as

(2) ρ(r,F)=ρ(0)(r)+∑jρj(1)(r)Fj+12!∑jρjk(2)(r)FjFk +12!∑jρjkl(3)(r)FjFkFl+⋯,ρj(1)(r)=∂ρ(r,F)∂Fj|Fj=0,αij=−∫ri  ρj(1)(r)dr.

**Figure 3:**
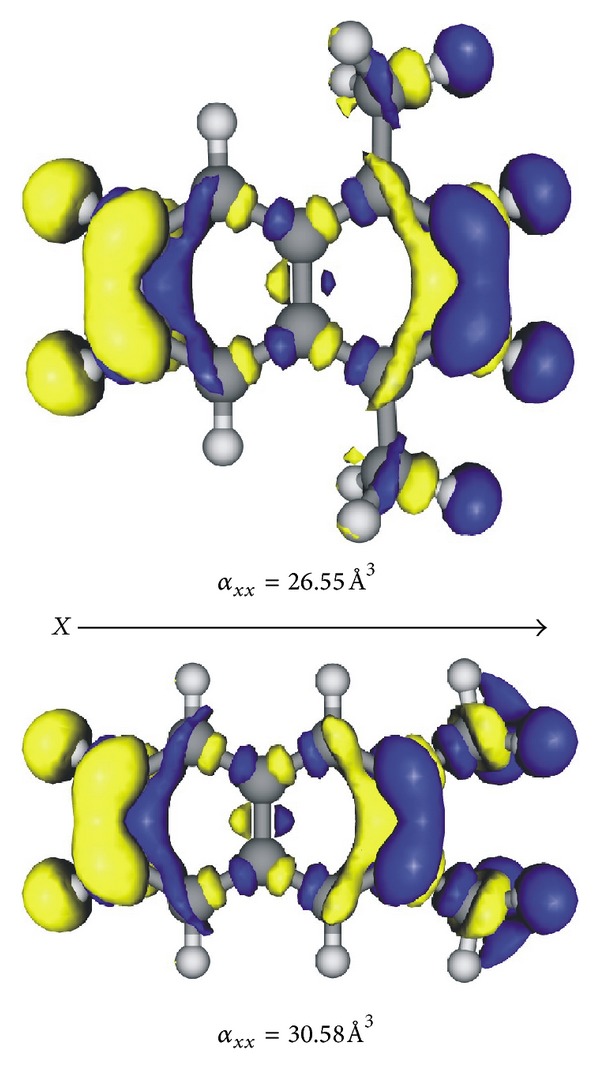
Calculated *ρ*_*x*_^(1)^(*r*) density distributions for the 1,4-DMN (top) and 2,3-DMN (bottom) isomers. Positive and negative are represented by yellow and blue isosurfaces (0.001 a.u.), respectively. CAM-B3LYP/6-31+G* results.

For a certain positive-negative *ρ*_*j*_^(1)^(*r*) pair, the sign is positive when the direction of the positive to negative densities coincides with the positive direction of the chosen coordinate system, whereas the magnitude is proportional to the distance between the two densities. In the present study, we determined the *ρ*_*j*_^(1)^(*r*) densities at the CAM-B3LYP/6-31+G* level for the longitudinal component (*j* = *x*). As representative cases of the *α*,*α*-DMN and *β*,*β*-DMN isomers, we investigated the *ρ*_*x*_^(1)^(*r*) densities for 1,4-DMN and 2,3-DMN. The *ρ*_*x*_^(1)^(*r*) distributions are displayed in Figure 3. From the CAM-B3LYP/6-31+G* computations, the *α*_*xx*_(2,3-DMN) value is higher than the corresponding datum of the 1,4-DMN isomer by about 4 Å^3^ (Table 2). For both the isomers, the largest positive to negative *ρ*_*x*_^(1)^(*r*) contributions are mainly placed over the N moiety. However, as can be appreciated from the plots in Figure 3, the 2,3-DMN isomer exhibits additional relevant *ρ*_*x*_^(1)^(*r*) densities localized on both the CH_3_ groups. These methylic *ρ*_*x*_^(1)^(*r*) amplitudes are much more conspicuous than those found for the 1,4-DMN isomer, contributing to the increase of the *α*_*xx*_ component by ca. 17%.

### 3.3. Static and Dynamic First-Order Hyperpolarizabilities of the DMN Isomers

The calculated static *β*_*xxx*_ and *β*_*vec*⁡_ values of the DMN isomers obtained in vacuum at the CAM-B3LYP/6-31+G* level are collected in Table 3. As for the computed dipole moments [14], due to the mutual disposition of the methyl substituents, the 1,5-DMN and 2,6-DMN isomers are nonpolar compounds. In the present study, besides to the static first-order hyperpolarizabilities we also determined the frequency-dependent properties for the SHG and EOPE NLO phenomena since observed data are nearly always obtained at incident optical fields. In order to minimize resonance enhancements, the dynamic hyperpolarizabilities were evaluated at the *λ* value of 1064 nm (*ℏω* = 0.04282 a.u.), which is sufficiently apart from the observed lowest-energy absorption of DMNs (the experimental *λ*_max⁡_ values are in the range 274–289 nm) [73]. The dynamic *β*_*vec*⁡_(−*ω*_*σ*_; *ω*_1_, *ω*_2_) data are included in Table 3. As should be expected, *β*_*vec*⁡_(−2*ω*; *ω*, *ω*)>*β*_*vec*⁡_(−*ω*; *ω*, 0) for all the isomers. The dispersion effects enhance the static *β*_*vec*⁡_(0; 0, 0) values by 6–18% and 17–57%, for the EOPE and SHG processes, respectively, the largest increases being found for 1,4-DMN, in agreement with its highest *λ*_max⁡_ value among the DMN isomers.

The static and dynamic *β*_*vec*⁡_ values concordantly predict the following order:

 1,4-DMN < 1,6-DMN < 2,7-DMN < 1,3-DMN < 1,7-DMN < 1,8-DMN < 1,2-DMN < 2,3-DMN.

Note that the above order is rather different from those obtained for the polarizabilities; a *β*_*vec*⁡_ versus *k*_*b*_ relationship cannot be established. In particular, the static and dynamic CAM-B3LYP/6-31+G*  *β*_*vec*⁡_(2,3-DMN) values are calculated to be 5-6 times higher than the corresponding data obtained for the 1,4-DMN isomer. More importantly, we notice that the first-order hyperpolarizabilities of the *β*,*β*-DMNs are somewhat different from each other for the 2,6-DMN isomer the *β*_*vec*⁡_ is zero since it belongs to the *C*_2*h*_ symmetry point group, whereas the *β*_*vec*⁡_(2,3-DMN)/*β*_*vec*⁡_(2,7-DMN) ratios are predicted to be 2.5–2.7 by the present static and dynamic computations. Similarly, the *β*_*vec*⁡_ values of the *α*,*α*-DMN isomers are rather different from each other. In fact, on passing from 1,4-DMN to 1,8-DMN, the *β*_*vec*⁡_(−*ω*_*σ*_; *ω*_1_, *ω*_2_)  data increase by a factor of three/four, whereas the *β*_*vec*⁡_(1,5-DMN) value is zero (*C*_2*h*_ symmetry point group). Note that a somewhat different situation occurs for the *α*,*β*-DMN isomers, which exhibit hyperpolarizabilities much closer to each other. 

Interestingly, as for the computed *β*_*vec*⁡_ data, *μ*(1,4-DMN) and *μ*(2,3-DMN) (Table 3) are, respectively, the smallest and greatest *μ* values along the series of the DMNs. The order of the *μ* values is roughly similar to that of the first-order hyperpolarizabilities, a linear relationship between the *β*_*vec*⁡_ and *μ* values being established (*β*_*vec*⁡_ = 118.32 + 948.6 × *μ*, *r* = 0.92). This result indicates that, as for the *μ* data, the *β*_*vec*⁡_ values of the DMNs are mainly affected by the mutual disposition of the CH_3_ groups, mesomeric effect enhancements of the hyperpolarizabilities being expected to be marginal.

As for the polarizabilities, we explored the hyperpolarizability differences between the 1,4-DMN and 2,3-DMN isomers through hyperpolarizability density analyses. The *β* density, *ρ* ^(2)^(*r*), is expressed as follows [71, 74]:

(3) $$\begin{array}{c} \rho_{jk}^{\left(2\right)}\left(r\right)={\frac{\partial^{2}\rho \left(r,F\right)}{\partial F_{j}\partial F_{k}}|}_{F_{j}=0,F_{k}=0},\\ \beta_{ijk}=-\frac{1}{2!}\int r\rho_{jk}^{\left(2\right)}\left(r\right)dr. \end{array}$$

**Figure 4:**
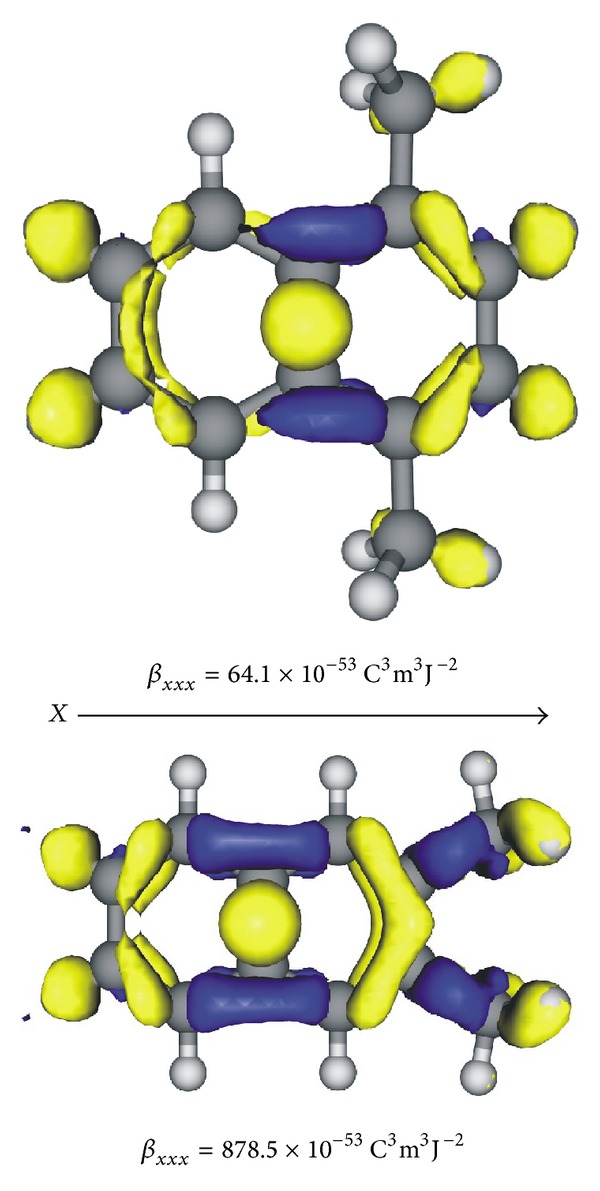
Calculated *ρ*_*xx*_^(2)^(*r*) density distributions for the 1,4-DMN (top) and 2,3-DMN (bottom) isomers. Positive and negative are represented by yellow and blue isosurfaces (0.001 a.u.), respectively. CAM-B3LYP/6-31+G* results.

By analyzing the main contributing components, for the 1,4-DMN isomer, the largest hyperpolarizability component lies along the *x*-axis. At the CAM-B3LYP/6-31+G*  level, the static *β*_*xxx*_(1,4-DMN) value is calculated to be 62.5 × 10^−53^ C^3^m^3^J^−2^, recovering ca. 35% of the *β*_*vec*⁡_(0; 0,0) value. Similarly, the *β*_*xxx*_ component dominates the first-order hyperpolarizability of 2,3-DMN, the *β*_*xxx*_(0; 0,0)/*β*_*vec*⁡_(0; 0,0) ratio being calculated to be 0.76. Thus, we computed the *ρ*_*jk*_^(2)^(*r*) distributions for the *xx* component at the CAM-B3LYP/6-31+G*  level using a numerical procedure described in detail in [74].  Figure 4 displays the *ρ*_*xx*_^(2)^(*r*) densities for the 1,4-DMN and 2,3-DMN isomers. As can be seen from the graphical representations, for both the isomers, the most significant *ρ*_*xx*_^(2)^(*r*) amplitude is furnished by the N skeleton, although it is much more expanded in the case of the 2,3-DMN isomer. In addition, different from the 1,4-DMN isomer, for 2,3-DMN, a conspicuous positive *ρ*_*xx*_^(2)^(*r*) contribution is also provided by both the CH_3_ groups. On the whole, the above *ρ*_*xx*_^(2)^(*r*) results are in qualitative agreement with the calculated static hyperpolarizabilities, the *β*_*xxx*_(2,3-DMN)/*β*_*xxx*_(1,4-DMN) and *β*_*vec*⁡_(2,3-DMN)/*β*_*vec*⁡_(1,4-DMN) ratios being predicted to be 14 and 6, respectively. 

## 4. Conclusions

In this study, we investigated the electronic polarizabilities and first-order hyperpolarizabilities of the series of DMN isomers. The computations were performed in vacuum using the CAM-B3LYP functional and the 6-31+G*  basis set. The response electric properties were obtained in the static and dynamic regimes for the SHG and EOPE NLO phenomena at the *λ* value of 1064 nm. The average polarizability varies a little along the series of isomers, whereas both the longitudinal polarizability and anisotropy of polarizability are much more affected by the position of the methyl substituents. In agreement with recent theoretical studies on the polarizabilities [14] and Raman spectra [15], linear relationships are established between the calculated polarizabilities and the experimental biodegradation rates of DMNs, confirming the important role of dispersive and/or inductive interactions for the biodegradative mechanisms of this group of substituted PAH isomers.

The static and frequency-dependent first-order hyperpolarizabilities are strongly dependent on the relative position of the CH_3_ groups. This is especially evident for the *α*,*α*-DMN and *β*,*β*-DMN isomers: 2,6-DMN < 2,7-DMN < 2,3-DMN and 1,5-DMN < 1,4-DMN < 1,8-DMN. The present results suggest that some DMN isomers might be distinguished through SHG and EOPE NLO measurements. Density analysis computations are useful for qualitative interpretations of the (hyper)polarizabilities of the DMN isomers. 

## Figures and Tables

**Table 1:** Static electronic 〈α〉 (Å^3^), Δ*α* (Å^3^), and *β*_*vec*⁡_ (10^−53^ C^3^m^3^J^−2^) of toluene^a^.

	〈α〉	Δ*α*	*β*_*vec*⁡_
CAM-B3LYP/POL	12.25	6.68	279.3
CAM-B3LYP/6-31+G*	11.50	6.43	316.6
MP2/6-31+G*	11.43	6.24	312.4

^a^All calculations are carried out on the B3LYP/6-31G* geometry.

**Table 2:** Static and dynamic (*ℏω* = 0.04282 a.u.) electronic α_*xx*_ (Å^3^), 〈*α*〉 (Å^3^) and Δ*α* (Å^3^) of the dimethylnaphthalene isomers and naphthalene^a^.

*ℏω*	*α*_*xx*_(−*ω*; *ω*)		〈*α*〉(−*ω*; *ω*)		Δ*α*(−*ω*; *ω*)		*k*_*b*_^b^
	0	0.04282	0	0.04282	0	0.04282	
1,2-DMN	28.53	29.14	20.65	21.00	15.00	15.46	0.100 ± 0.03
1,3-DMN	28.74	29.36	20.77	21.12	15.03	15.50	0.120 ± 0.04
1,4-DMN	26.55	27.09	20.54	20.89	13.80	14.20	0.055 ± 0.02
1,5-DMN	26.58	27.12	20.55	20.90	13.89	14.30	0.065 ± 0.01
1,6-DMN	28.76	29.38	20.78	21.13	15.19	15.65	0.120 ± 0.04
1,7-DMN	28.84	29.46	20.76	21.11	15.08	15.55	
1,8-DMN	26.77	27.32	20.42	20.76	13.97	14.38	0.033 ± 0.02
2,3-DMN	30.58	31.27	20.90	21.25	16.55	17.08	
2,6-DMN	31.06	31.76	21.07	21.43	17.12	17.67	0.200 ± 0.05
2,7-DMN	31.09	31.79	21.06	21.42	16.90	17.45	0.360 ± 0.07
N	24.20(24.39)^c^		16.91(17.40)^c^		13.21(12.91)^c^		

^a^All calculations were carried out at the CAM-B3LYP/6-31+G* level on the B3LYP/6-31G* geometry taken from [14].

^b^Experimental first-order biomass-normalized rate coefficient *k*_*b*_ ((mg of protein/L)^−1^(h)^−1^) in aqueous systems, [12].

^c^[70].

**Table 3:** Calculated *μ*(*D*), static and dynamic (*ℏω* = 0.04282 a.u.) electronic *β*_*xxx*_(0; 0,0) and *β*_*vec*⁡_(−*ω*_*σ*_; *ω*_1_, *ω*_2_) (10^−53^ C^3^m^3^J^−2^) of the dimethylnaphthalene isomers^a^.

	*μ*	*β*_*xxx*_(0; 0,0)	*β*_*vec*⁡_(0; 0,0)	*β*_*vec*⁡_(−*ω*; *ω*, 0)	*β*_*vec*⁡_(−2*ω*; *ω*, *ω*)
1,2-DMN	0.69	430.3	743.9	808.0	923.4
1,3-DMN	0.54	−412.0	599.6	660.5	782.3
1,4-DMN	0.06	62.5	179.6	211.6	282.2
1,5-DMN	0.00	0.0	0.0	0.0	0.0
1,6-DMN	0.49	−348.8	448.9	474.5	529.0
1,7-DMN	0.62	370.3	628.4	670.1	737.5
1,8-DMN	0.66	0.0	673.3	734.2	859.3
2,3-DMN	0.90	879.8	1157.5	1256.9	1474.9
2,6-DMN	0.00	0.0	0.0	0.0	0.0
2,7-DMN	0.20	0.0	461.7	497.0	541.9

^a^All calculations were carried out at the CAM-B3LYP/6-31+G* level on the B3LYP/6-31G* geometry.
